# The Interface between BCR-ABL-Dependent and -Independent Resistance Signaling Pathways in Chronic Myeloid Leukemia

**DOI:** 10.1155/2012/671702

**Published:** 2012-04-24

**Authors:** Gabriela Nestal de Moraes, Paloma Silva Souza, Fernanda Casal de Faria Costas, Flavia Cunha Vasconcelos, Flaviana Ruade Souza Reis, Raquel Ciuvalschi Maia

**Affiliations:** Laboratório de Hemato-Oncologia Celular e Molecular, Programa de Pesquisa em Hemato-Oncologia Molecular, Instituto Nacional de Câncer (INCA), Praça da Cruz Vermelha 23, 6° andar, Centro, 20230-130 Rio de Janeiro, RJ, Brazil

## Abstract

Chronic myeloid leukemia (CML) is a clonal hematopoietic disorder characterized by the presence of the Philadelphia chromosome which resulted from the reciprocal translocation between chromosomes 9 and 22. The pathogenesis of CML involves the constitutive activation of the BCR-ABL tyrosine kinase, which governs malignant disease by activating multiple signal transduction pathways. The BCR-ABL kinase inhibitor, imatinib, is the front-line treatment for CML, but the emergence of imatinib resistance and other tyrosine kinase inhibitors (TKIs) has called attention for additional resistance mechanisms and has led to the search for alternative drug treatments. In this paper, we discuss our current understanding of mechanisms, related or unrelated to BCR-ABL, which have been shown to account for chemoresistance and treatment failure. We focus on the potential role of the influx and efflux transporters, the inhibitor of apoptosis proteins, and transcription factor-mediated signals as feasible molecular targets to overcome the development of TKIs resistance in CML.

## 1. Introduction

Chronic myeloid leukemia (CML) is a myeloproliferative disorder that results from the reciprocal translocation of the *ABL1* oncogene on chromosome 9 with the breakpoint cluster region (*BCR*) gene on chromosome 22 [*t*(9; 22)], leading to the formation of the BCR-ABL oncoprotein. The shortened chromosome 22 formed by this translocation is the Philadelphia (Ph) chromosome. The *BCR-ABL* fusion oncogene, which is responsible for the pathogenesis of CML, has greatly enhanced ABL1 tyrosine kinase constitutive activity [1]. CML is characterized by a biphasic evolutive course. Most patients are diagnosed in the chronic phase (CML-CP), which is characterized by the absence of symptoms in half of the patients. However, a prominent leukocytosis is frequently observed by routine testing. In the other half of patients, symptoms are common and include splenomegaly, weight loss, lethargy, and anemia [2]. The disease may progress either directly to blast phase (BP) or through an intermediate accelerated phase (AP). The time course for progression to BP is variable and the molecular mechanisms underlying disease progression are extremely complex. BCR-ABL-dependent pathways to blast transformation include an increase in genomic instability, telomere shortening, loss of tumor-suppressor function, and inhibition of tumor suppressors with cell regulatory functions [2, 3].

In order to identify prognostic factors for CML patients, many clinical and biological characteristics have been analyzed. Sokal risk score (based on spleen size, age, platelet count, and peripheral blood blast) is a prognostic factor widely used for prediction of cytogenetic response and of progression-free and overall survival in CML-CP with imatinib as front-line therapy. Other factor predictors for therapy response include OCT-1 activity, *ABCB1*/P-glycoprotein overexpression and polymorphisms, *in vivo* measurement of the Crkl phosphorylation, and molecular response [4].

 The treatment of CML-CP can be divided into pre-imatinib and post-imatinib era. Prior to the imatinib era, busulphan and interferon-*α* recombinant [5, 6] were used to control and to prolong CML survival in the CP phase, but allogenic stem-cell transplantation was, and is still, the only therapy with potential for curing CML patients [7]. After the introduction of imatinib, a potent tyrosine kinase inhibitor (TKI), there was a dramatic change in the CML outcome. Imatinib acts by binding to the BCR-ABL protein in the inactive conformation and is unable to bind to the active configuration [8]. The survival rate attributed to imatinib is arguably more elevated than interferon-based therapy [9]. In addition, imatinib is generally well tolerated [10]. Imatinib treatment is associated with high rates of complete cytogenetic and major molecular responses in patients with CML-CP. On the other hand, despite improvements related to survival by using imatinib or other TKIs, CML-BP prognosis remains disappointing [11].

Currently, imatinib is the standard therapy for all CML phases [12–14]. Despite the clinical success with imatinib demonstrating long-term survival for the majority of patients, one-third of patients need an alternative therapy, frequently a second-generation TKI, such as dasatinib and nilotinib. Patients who need second-line therapy include those with imatinib intolerance [10] or mainly primary or acquired imatinib resistance [15, 16].

The most common mechanism of resistance to imatinib is the development of point mutations or amplification of the *BCR-ABL *gene, which alters the kinase domain (KD) of BCR-ABL and is responsible for imatinib loss of efficacy [17]. KD mutations can be found at any phase of CML. Not all KD mutations are responsible for TKI resistance. However, T315I mutation is generally resistant to all TKIs [18].

 BCR-ABL acts with other multiple cellular and genetic events that accumulate progressively to drive the disease into the blast phase. Therefore, additional mechanisms—dependent or independent to BCR-ABL—may also account for resistance to imatinib treatment and result in a poor outcome. In this review, the role of efflux and influx transporters, inhibitor of apoptosis proteins (IAP), and transcription factors as additional mechanisms responsible for chemoresistance in CML will be discussed.

### 1.1. Efflux and Influx Transporters

The multidrug resistance (MDR) phenotype related to increased expression of efflux pumps, such as ABCB1/P-glycoprotein (Pgp) and ABCG2/breast-cancer-related protein (BCRP), is one of the most studied mechanisms of resistance in CML. More recently, the decrease in influx transporters, such as the organic cation transporter-1 (Oct-1), has also emerged as a mechanism responsible for inefficient drug uptake and consequent treatment failure [16, 19].

#### 1.1.1. ABCB1/P-Glycoprotein

The most common mechanism developed by tumor cells to escape a drug-induced death is displayed in intrinsic or acquired MDR phenotype by the overexpression of the drug-efflux protein ABCB1 [20, 21]. ABCB1, a product of the *ABCB1* gene, was first described in 1976 by Juliano and Ling, who observed a cell surface glycoprotein that altered drug permeability in hamster drug-resistant cells. Human cells also express ABCB1 on the cell surface, acting as a drug efflux pump and, consequently, decreasing intracellular drug concentration [22, 23]. Meanwhile, physiological ABCB1 expression has been identified in some tissues, particularly on the membranes of kidney tubules, in the canalicular membranes of hepatocytes, in the gastrointestinal tract, at blood tissue barriers, in the placenta, and in blood cells including CD34^+^ hematopoietic stem cells, natural killer cells, antigen-presenting dendritic cells (DC), and T and B lymphocytes [24–28]. Its physiological function suggests a protection against potentially toxic compounds and harmful substances found in the blood stream. Studies on ABCB1 knockout mice showed no physiological abnormalities under normal conditions, although these animals display hypersensitivity to drugs and an increase in ABCB1 substrate accumulation [27, 29–31]. 

Clinical insensitivity to anticancer agents is mainly attributed to an elevated expression of ABCB1, which is related to treatment failure associated with lower remission and survival rates in some types of cancer, including leukemias [32–34]. Meanwhile, gene and protein expressions of ABCB1 are commonly acquired or increased during the course of chemotherapy, which make drug treatment a responsible factor for MDR [35, 36]. Other extrinsic factors may induce MDR by acquisition of ABCB1 expression. Levchenko et al. [37] showed that ABCB1, and, consequently, MDR are transferred by direct membrane contact of tumor cells. Moreover, resistant tumor cells may release membrane microparticles carrying surface ABCB1. The shared microparticles can bind to receptor cells, spread ABCB1 and, consequently, induce MDR phenotype [38].

Even though the ABCB1 efflux functions, other functions for this transporter have been studied. Studies have shown that the resistance induced by ABCB1 is also associated with the inhibition of cell death, and ABCB1 promotes additional protection to caspases-dependent apoptosis, UV radiation, serum starvation condition, and spontaneous apoptosis [39–42]. Recently, our group demonstrated that ABCB1 expression induced by drug treatment promotes resistance to apoptosis in BCR-ABL cells independently of its drug-efflux activity [43].

 ABCB1 is related to resistance phenotype in some leukemias and it has been studied in advanced CML. A randomized trial evaluated the relevance of ABCB1 expression in CML patients. The authors observed that the response to cytarabine and daunorubicin was significantly related with both ABCB1 expression and function mainly in the blast phase. For this reason, chemotherapy resistance in CML-BP patients should be considered multifactorial and cannot be associated only with BCR-ABL [44–47]. Our group recently demonstrated that CML patients show high levels of ABCB1 expression independently of CML phases. Nevertheless, we showed that ABCB1 expression is more frequent than multidrug-resistant protein 1 (MRP1) in CML-BP [48].

*In vitro* data suggest that imatinib is able to induce ABCB1 in sensitive CML cell lines and, as a result, ABCB1 activity may confer resistance to this drug [49–51]. Mahon et al. [52] demonstrated that a multidrug-resistant CML cell line displayed resistance to many drugs including imatinib and the induced overexpression of *ABCB1* gene by retroviral transduction in BCR-ABL cell line also leads to imatinib resistance. Moreover, Rumpold et al., [53] showed that a stable silencing of ABCB1 in imatinib-resistant CML cell lines abolished ABCB1-efflux substrates and induced sensibility to imatinib. Regardless of the *in vitro *data, there is no consistent evidence for this resistance *in vivo*, although several studies have discussed the role of ABCB1 in imatinib-resistant CML patients [54]. Zong et al. [55] demonstrated that bone marrow mice cells Mdr1a/1b-null transduced with *BCR-ABL *display a similar response to imatinib, which is related to increased peripheral white blood cells counts and marked hepatosplenomegaly, compared with *BCR-ABL*-transduced wild-type bone marrow. The authors concluded that the expression of ABCB1 in hematopoietic stem cells does not interfere with imatinib resistance. Another *in vivo* study revealed that imatinib treatment in CML patients in the accelerated phase induced an increase of ABCB1-positive cells with efflux activity. However, in imatinib-resistant CML patients, the efflux activity was independent of ABCB1 expression, suggesting participation of other ABC transporters [56]. Hatziieremia et al. [57] inhibited ABCB1 using PSC833 in CD34^+^ cells from CML-CP patients and did not observe imatinib efficiency in eliminating CML cells.

 Although these previously described works do not identify the role of ABCB1 in imatinib resistance, studies in polymorphisms of ABCB1 have shown the importance of ABCB1 in CML treatment resistance. Moreover, this kind of study may provide information for the prediction of drug disposition in a specific way and promote better response to imatinib in CML patients [58, 59]. Dulucq et al. [60] analyzed 1236C>T, 2677G>T/A, and 3435C>T ABCB1 single nucleotide polymorphisms (SNPs) in CML patients treated with imatinib. The authors observed that allele G in 2677G>T/A polymorphism was associated with the worst response to imatinib. In a Chinese population, Ni et al. [61] observed more imatinib resistance in patients homozygous for 1236T allele and 3435 TT/CT genotypes.

Studies have suggested that second- and third-generation TKIs can overcome imatinib resistance [62, 63]. There are studies suggesting that nilotinib does not induce resistance in CML cells through ABCB1 overexpression [64]. Nevertheless, Mahon et al. [65] developed nilotinib-resistant CML cell lines and observed that nilotinib is a substrate for ABCB1. Moreover, concomitant overexpression of ABCB1 and BCR-ABL provides nilotinib resistance in CML cells. Studies also revealed the interaction of dasatinib and ABCB1 efflux protein. Giannoudis et al. [66] showed that cell lines BCR-ABL (positive or not) are able to extrude dasatinib through ABCB1 activity. In concordance, Hiwase et al. [67] demonstrated that ABCB1 is able to transport dasatinib from CML cells. These studies show the importance of researching more about ABCB1 expression, function, and inhibition.

An important strategy to try reversing clinical MDR involves modulation or inhibition of ABCB1. The cyclosporine A (CsA) is capable of regulating the efflux function of ABCB1 dependently on its concentration in cancer cells [68, 69]. Some studies in hematological cancer have shown the benefits of CsA on reversing MDR or potentiating drug effects [70]. In a clinical study of our group, we evaluated the effect of CsA on the circumvention of leukemia patients MDR *in vitro*. Our data showed that combination of CsA and etoposide (VP-16) could induce a good response in ABCB1-positive CML patients [71]. In the same year, we also published a case report showing that the cytotoxic effect of VP-16 was enhanced in combination with CsA in blast cells of CML. Moreover, the patient returned from blast phase to chronic phase [72]. All these studies and others emphasized the importance of reversing the MDR phenotype.

#### 1.1.2. BCRP/ABCG2

Another important efflux pump associated with chemotherapy resistance in CML is BCRP or ABCG2, coded by the gene *ABCG2*. ABCG2 is a 72-kDa protein composed of 665 amino acids. It has an N-terminal ATP-binding domain (NBF) and a C-terminal transmembrane domain (TMD), a structure half the size and in reverse configuration to most other ABC proteins comprising two NBFs and two TMDs. Because ABCG2 is a half-transporter, it is believed to homodimerize, or possibly oligomerize, in order to function [73].

Fetsch et al., [74] reported high levels of ABCG2 expression in normal placenta, interstitial cells of testes, endocervical cells of uterus, squamous epithelium of cervix, kidney, hepatocytes, pancreas, and small and large intestinal mucosa/epithelial cells. The first reported chemotherapy agent substrate of ABCG2 was mitoxantrone [75]. Other chemotherapeutic substrates include flavopiridol, topotecan, methotrexate, and the TKIs imatinib, gefitinib, and erlotinib [76]. If the amino acid at position 482 is mutated, mitoxantrone transport is more efficient and ABCG2 can additionally transport rhodamine 123 and anthracyclines such as doxorubicin and bisantrene [77, 78].

It was demonstrated that TKI had high-affinity interaction with ABCG2 and that it occurs at submicromolar concentrations [79, 80]. Although other TKIs promote ATPase activity, imatinib was the only one able to inhibit it, suggesting that this drug acts as a modulator agent. In addition, imatinib has promoted the accumulation of a fluorescent substrate inside the cells which reinforced its role as a modulator. Using a different methodology, Houghton et al. [81] demonstrated that overexpression of ABCG2 was not able to confer resistance to imatinib, suggesting that it is not a substrate for this transporter. In addition, imatinib promoted the accumulation of topotecan in functional ABCG2-expressing cell lines, indicating a role of imatinib as a modulator but not as a competitor. Conversely, Ko-143, an ABCG2 specific modulator, could increase the imatinib accumulation in ABCG2-overexpressing cell lines, suggesting its role as a competitor. Interestingly, mitoxantrone accumulation in the same cell lines was increased by the addition of imatinib, suggesting its role as a modulator. These findings suggest that imatinib can be both a substrate and a modulator [82]. Furthermore, Brendel et al. [83] confirmed that ABCG2 expression confers imatinib resistance and reduces imatinib accumulation in K562 cells, effects that are abrogated by the ABCG2 inhibitor fumitremorgin C (FTC). More importantly they observed that differences on imatinib accumulation were only seen when imatinib was used at low concentrations but not at high concentrations. These data support the idea that imatinib may act as a modulator or a substrate depending on the concentration level. However, there is still no consensus on whether imatinib is a substrate or a modulator of ABCG2 transport. Regardless of its role as a substrate or modulator, imatinib interacts with ABCG2, which may have their effectiveness limited by the overexpression of this protein.

It is believed that CML is a clonal disorder originating from the hematopoietic stem cell (HSC). Graham et al. [84] demonstrated that primitive HSCs expressing BCR-ABL in CML-CP patients were resistant to imatinib. This discovery led to the hypothesis that the resistant population could contribute to the failure of treatment with imatinib. Nakanishi et al. [85] studied the interaction of ABCG2 and imatinib on a cell line expressing BCR-ABL. This cell line was resistant to substrates of ABCG2 as well as imatinib, and the resistance was reversed by inhibiting ABCG2. Another interesting finding was that the initial resistance to imatinib caused by ABCG2 was attenuated by the inhibition of BCR-ABL, suggesting that BCR-ABL regulates the expression of ABCG2 at a later stage of transcription.

Some authors have identified the presence of ABCG2 in a particular group of HSC called “side population” (SP) due to its efflux of the fluorochrome Hoechst 33342 and its ability to reconstitute bone marrow in irradiated mice [86]. Afterward, it was demonstrated that ABCG2 was responsible for the SP in mouse and human bone marrow [87, 88]. *ABCG2*-deficient mice are viable with normal numbers of stem cells. Despite the absence of SP, these data suggest that ABCG2 protein is not necessary for normal hematopoiesis [89]. However, ABCG2 may play a protective role for stem cells, because Zhou et al. [90] demonstrated that stem cells derived from *ABCG2*-deficient mice were more sensitive to cytotoxic substrates.

Once ABCG2 is expressed in the apical membrane of cells in the epithelium of the small intestine and colon, it is very likely that ABCG2 is involved in the active return of drug entering the intestine. This role would be important in reducing the systemic bioavailability of oral drugs such as imatinib. Studies in ABCG2 knockout mice indicate that ABCG2 and ABCB1 appear to regulate the penetration of imatinib into the brain tissue. Imatinib brain penetration in ABCG2 knockout mice was found to be increased [91].

The SNP 421C>A is responsible for decreased plasma membrane expression of ABCG2, reduced ATPase activity, or decreased drug transport [92–94]. Therefore, the daily imatinib dose for patients with the *ABCG2* 421C/C genotype might be higher than for those with the 421C/A or 421A/A genotype [95]. Knowledge of the *ABCG2* 421 genotype could be useful when making dosing decisions aimed at achieving the optimal imatinib exposure.

### 1.2. SLC22A1/OCT-1 Influx Transporter Protein

Members of the solute carriers (SLCs) superfamily of transporters are known as passive facilitator carriers that allow the passage of solute through the membrane without spending energy [96]. This superfamily is divided into 43 families according to the type of substrate transported and the type of transport. Some families carry specific substrates such as oligopeptides, sugars, phosphatases, or metals, whereas other families are polyspecific, transporting substrates with different sizes and structures [97].

 SLC transporters are mostly expressed in the plasma membrane and play a critical role in a variety of physiological cellular processes such as import/export neurotransmitters, nutrients, or metabolites [96]. The family 22 of solute carrier proteins is composed of 12 members mostly of poly-specific transporters. Many members of this family are expressed in the intestine, liver, and kidney, indicating an important role in the absorption and excretion of drugs, xenobiotics, and endogenous compounds that exist as cations at physiological pH. The family is further divided into subgroups according to the substrate and the transport mechanism [96].

 A growing number of scientific papers have shown that some chemotherapeutics are substrates for influx transporters. Recently, it was reported that imatinib is transported into the cell, preferably via SLC22A1 (also called OCT-1), and the expression of this transporter is predictive of achieving a complete cytogenetic remission after 6 months of treatment with imatinib [98].

 It was reported that the influx of imatinib is temperature dependent, indicating the involvement of an active process of influence. When the cells were incubated with inhibitors of the transporter SLC22A1, the influx of imatinib was significantly reduced [99]. Since then, other studies have been published supporting the hypothesis that imatinib is a substrate for the transporter SLC22A1. White et al. [100] analyzed the activity of SLC22A1 in samples from CML patients before starting treatment with imatinib and compared this with getting a major molecular response at 24 months. In this study, the activity of SLC22A1 was an important determinant of molecular response to imatinib with strong predictive value on the dose. The analysis of SLC22A1 activity before the start of treatment with imatinib was able to identify patients who would need a higher dose of the drug to respond to medical treatment with imatinib.

 Besides the SLC22A1 activity, the levels of expression of SLC22A1 may be related to a decreased influx of imatinib. Crossman et al. [101] analyzed the expression of the *SLC22A1* gene in samples from CML patients before starting treatment and observed that the expression of *SLC22A1* was variable and did not differ significantly from levels found in samples of bone marrow healthy individuals. However, patients who responded to treatment with imatinib had significantly higher levels of expression of the *SLC22A1* gene as compared to the group of nonresponders. Despite this and other articles suggesting a direct correlation between SLC22A1 and response to treatment with imatinib, Hu et al. [102] believe that SLC22A1 *per se* is not able to influence the retention of imatinib, as this drug would be a poor substrate for SLC22A1. On the other hand, Wang et al. [98] suggested that clinical responses to imatinib could be affected by transporters SLC22A1, ABCB1, and ABCG2. Patients with high pretreatment SLC22A1 expression had a higher probability of achieving a cytogenetic response and a superior progression-free and overall survival. The same was not observed when analyzing ABCB1 and ABCG2.

The contribution of the SLC22A1 transporter to the clinical response to imatinib has not yet been elucidated. Therefore, further studies are needed to evaluate the role of this influx transporter in the clinical outcome of imatinib treatment.

## 2. Inhibitor of Apoptosis Proteins (IAPs)

The IAP family members are characterized by a common baculoviral IAP repeat (BIR) domain [103] and by the ability to block apoptosis through the inhibition of both mitochondrial-dependent and -independent apoptotic pathways [104, 105]. Among IAPs, much attention has been focused on survivin and XIAP due to their potential role as therapeutic targets.

### 2.1. XIAP

XIAP (X-linked of inhibitor of apoptosis protein) is a singular IAP because it is the only member of the family known to directly inhibit caspases-3, -7, and -9 [106, 107]. XIAP is able to bind their target caspases by a two-site interaction mechanism, which inhibits the apoptotic pathway by blocking the active caspase site or by dissociating the dimer of caspases [108].

There are at least two proteins, Smac/DIABLO [109] and XAF1 (XIAP-associated factor 1) [110], known to interact with XIAP and modulate its antiapoptotic activity, which suggests a significant role of XIAP in the maintenance of the cellular homeostasis [111]. Other relevant XIAP properties are the involvement in copper metabolism [112] and the capacity of self-ubiquitination and of other targets involved or not in the control of the cell death [113], demonstrating its versatility in the cellular physiologic processes. Studies using knockout murine models for XIAP (*XIAP/BIRC4* ^−/−^) showed that its absence does not alter caspases-dependent or -independent apoptosis, but increases the expression of other IAPs, possibly as a compensatory mechanism [114].

XIAP is widely expressed in normal tissues [115]; however, its overexpression in cancer is usually associated with an unfavorable prognosis [116–119]. Although it has been demonstrated that the nuclear localization of XIAP is an independent prognostic marker in breast cancer [120], little is known about the expression and subcellular localization relevance of XIAP in CML patient samples.

Increasing evidence demonstrates that treatment of CML cells with chemotherapeutic agents can overcome resistance through negatively regulating XIAP levels. Fang et al. [121] have observed that one of the mechanisms involved in BCR-ABL-positive cells sensitivity to imatinib is XIAP downregulation. Corroborating this data, a study conducted in K562 cells and leukemic blasts obtained from patients with CML in blast crisis showed that apicidin, a histone deacetylase inhibitor, was able to potentiate imatinib effects on apoptosis through XIAP degradation and the release of the proapoptotic protein Smac/DIABLO into cytosol [122]. These events were associated with reduced BCR-Abl protein expression and decreased phosphorylated Akt levels and were caspase dependent [122]. Imatinib-induced apoptosis could also be potentiated when coadministered with ABT-737, a Bcl-2 and Bcl-xL inhibitor [123]. Cotreatment of K562 cells and primary CML samples led to caspase-3 activation and HtrA2/Omi-mediated decreased XIAP levels both in K562 cells and TKI-insensitive CML hematopoietic progenitors [123]. In addition to these findings, treatment of K562 cells with TRAIL led to an apoptosis-resistant phenotype through the upregulation of antiapoptotic proteins, including XIAP [124], further emphasizing its role in chemoresistance in CML.

Many strategies have been used to inhibit both the expression and function of XIAP and resensitize cancer cells to different cytotoxic stimuli [125–127]. One study demonstrated that the downregulation of XIAP expression using antisense oligonucleotides increased the sensitivity to cytotoxic stimuli, inducing apoptosis and decreasing cell viability in the K562 cell line [128]. Recently, the same group showed that the simultaneous inhibition of XIAP and P-glycoprotein in cells that overexpress this efflux pump decreases imatinib resistance [129]. Consistent with this, a recent work published by our group found that cyclosporine-A-mediated Pgp modulation was associated with XIAP inhibition and an increased apoptotic index as a response of resistant CML cells to vincristine [130]. Altogether, these findings point XIAP as an interesting therapeutic target and suggest that combining chemotherapeutic agents with XIAP-targeted therapy seems to represent a promising strategy in CML.

### 2.2. Survivin

Survivin, another IAP member, is an antiapoptotic protein [131], which also regulates cell division by controlling mitotic spindle checkpoint [132]. *Survivin* gene generates five different splice variant mRNAs, which encodes different proteins: wild-type survivin, survivin-2B, survivin-3B, survivin-*δ*Ex3, and survivin-2*α* [133]. Compared to wild-type survivin, little prognostic information is known about the functions of alternative splicing forms, which are generally expressed at lower levels than the wild-type survivin. In a recent study, it was found that patients in blast and accelerated phases displayed significantly lower levels of survivin-2B and -*δ*Ex3, compared to patients in CML-CP. However, there was no correlation between the isoform expression and clinical parameters or response to imatinib treatment [134].

Undetectable in normal differentiated tissues, survivin is abundantly expressed in all the most common human cancers [131, 135], which makes this protein a potential target for drug discovery and new anticancer interventions. Survivin can also be found in normal tissues characterized by self-renewal and proliferation [136], but its expression is significantly lower than in tumor cells. In CD34^+^ hematopoietic progenitor stem cells, survivin was found to be expressed and associated with the inhibition of apoptosis [137]. However, a recent report showed that despite survivin being quite expressed in CD34^+^ cells, its levels are low in more precursor leukemia stem cells [138], indicating that survivin is not an optimal therapeutic target for CML stem cells compartment and suggesting that it may not be the main factor accounting for resistance to targeted therapy in CML [139].

 In CML patient samples, several studies have reported that survivin was expressed in the accelerated and blast phases but it was low or undetectable in the chronic phase [4, 140–143], suggesting that survivin may be involved in the pathogenesis of progression from the CML-CP to the CML-BP. In addition, survivin overexpression in CML patients was correlated with the percentage of Ph chromosome positive cells and BCR-ABL expression [142], indicating that it can be regulated by BCR-ABL tyrosine kinase. In fact, Carter et al. [144] demonstrated that BCR-ABL and its downstream effector mitogen-activated protein kinase (MAPK) could target survivin expression at both RNA and protein levels in cells derived from a patient with CML-BP Ph chromosome positive. Survivin downregulation resulted in reduced cell viability in imatinib-sensitive CML cells, but not in imatinib-resistant CML cells or Ph chromosome negative cells, showing that survivin is regulated by the BCR-ABL/MAPK cascade in Ph positive CML. The prognostic importance of survivin in CML was also evaluated in a study from our group, where a correlation between survivin highest levels and high/intermediate Sokal score patients could be observed [145]. In addition, it was reported that survivin overexpression at diagnosis correlated with a low probability to achieve an optimal response to imatinib [134]. These data suggest that survivin may be closely involved in a more aggressive evolution of CML.

Growing evidence suggests that survivin plays an important role in chemoresistance phenotype of human malignancies [146], including CML. It has been demonstrated by our group that treatment of K562 CML cells with imatinib resulted in survivin downregulation and cell death [147]. Consistent with this, imatinib-induced apoptosis was increased when survivin expression was disrupted in BCR-ABL cells, as shown by enhanced cytochrome *c* release, caspase-9 activity, and BCR-ABL cleavage 199, which indicate that targeting survivin might be a useful tool to sensitize BCR-ABL cells to imatinib. Survivin has also been shown to play a resistant factor to agents other than imatinib in CML cells. In a recent publication, our group showed that survivin overexpression was involved in the resistance to idarubicin, an anthracycline commonly used to treat acute leukemia. On the other hand, idarubicin could induce DNA fragmentation and caspase-mediated apoptosis in K562 cells when survivin levels were down-regulated [148]. In addition, other groups have demonstrated that survivin inhibition is a common mechanism of apoptosis induced in CML cells by different classes of anticancer agents such as aurora kinase inhibitors, histone deacetylase inhibitors [149], microtubule targeting agents (MTAs), and cyclin-dependent kinase (CDK1) inhibitors [150]. Altogether, this amount of data shows that the modulation of survivin expression seems to be an interesting approach to overcome resistance and induce cell death in CML cells.

 In recent years, considerable efforts have been made to validate survivin as a new target in cancer therapy. YM-155, a small-molecule inhibitor of survivin, was the first survivin-targeted therapy to be developed and tested in clinical trials. In CML, YM-155 anticancer efficacy has been recently assessed in a preclinical study, where CML-derived cell lines showed great sensitivity to the molecule [151]. This effect has also been demonstrated for sheperdin, which is a novel antagonist of the interaction between hsp90 and survivin, known to be important for stabilizing survivin cytoprotective functions [152]. Although sheperdin did not decrease the viability of phytohemagglutinin-stimulated peripheral blood mononuclear cells or induced organ toxicity in a xenograft acute myeloid leukemia (AML) model, it could inhibit viability in K562 cells and in patient-derived AML peripheral blasts [153], demonstrating that it is a highly selective molecule. Antisurvivin therapies developed, to date, have not revealed major systemic toxicities in animal models and clinical trials and are extremely encouraging. Targeting survivin alone or in conjunction with chemotherapeutic agents has a great potential as a novel therapeutic regimen in CML.

## 3. Transcription Factors

Signal transduction pathways within the cell act by transmitting the extracellular signals to transcription factors, which result in changes in gene expression. However, it is well known that most key signaling pathways are deregulated in cancer, leading to altered expression and function of transcription factors. The constitutive activation of the nuclear factor kappa B (NF*κ*B) [169] and the inactivation of the forkhead box O (FoxO) factors [170] were shown to be important steps in carcinogenic transformation. Therefore, modulating the activity of FoxO and NF*κ*B seems to represent a reasonable therapeutic strategy.

### 3.1. NF*κ*B

Nuclear Factor *κ*B (NF*κ*B) was discovered in 1986 as a factor in the nucleus of B cells that bind to the enhancer of the kappa light chain of immunoglobulin [171]. It has been shown to be expressed in the cytoplasm of all cell types and, once activated, it translocates to the nucleus, where it regulates the expression of over 200 genes [172]. NF*κ*B is an important transcription factor typically activated by proinflammatory cytokines and other specific stimuli, and is involved in the regulation of a variety of biological responses, such as inflammatory, apoptotic, and immune processes. It achieves this by regulating the expression of proteins such as cytokines, chemokines, adhesion molecules, and the cellular death cascade [173]. The members of NF*κ*B protein family form dimers (usually heterodimers of p50 and p65 subunits) that interact in the cytoplasm with inhibitor of NF*κ*B (I*κ*B) proteins. When I*κ*B is phosphorylated by I*κ*B kinases (IKKs), it is degraded by the ubiquitin-proteasome pathway, liberating NF*κ*B dimers from their inhibition and allowing them to migrate to the nucleus and to activate NF*κ*B target genes [174].

In addition to its function as a central mediator of human immune responses, NF*κ*B plays a major role in activating genes involved in cellular survival, transformation, and oncogenesis. Loss of the normal regulation of NF*κ*B has become apparent as a major contributor to the deregulated growth, resistance to apoptosis, and propensity to metastasize observed in many cancers [175]. The overexpression of p65- or c-Rel-containing dimers can impair apoptosis, whereas the inhibition of NF*κ*B/Rel activity can enhance death induced by TNF-alpha, ionizing radiations, or chemotherapeutic agents in many cell types. Aberrant activation of NF*κ*B/Rel factors contributes to reduce the sensitivity to apoptosis in a vast range of hematologic malignancies. Although alterations in *NF*κ*B* or *I*κ*B* genes are documented in some neoplasms, in other cases, dysfunctions in components of the NF*κ*B/Rel-activating signaling pathways or influences of other mutated proteins on NF*κ*B/Rel can be recognized [176]. Constitutively active NF*κ*B has been detected in malignant cells derived from patients with multiple myeloma, AML, ALL, CML, and, most recently, in myelodysplastic syndromes. Targeting NF*κ*B in these hematopoietic malignancies leads to apoptosis, corroborating the role of NF*κ*B in the survival and clonal expansion of malignant cells [174].

The expression of BCR-ABL leads to the activation of NF*κ*B-dependent transcription by causing nuclear translocation of NF*κ*B and by increasing the transactivation function of the RelA/p65 subunit of NF*κ*B. Importantly, this activation is dependent on the tyrosine kinase activity of BCR-ABL that partially requires Ras. It has also been demonstrated that NF*κ*B is required for BCR-ABL-mediated tumorigenicity in nude mice and for transformation of primary bone marrow cells [169]. This activation regulates the transcription of important genes, such as c-myc, which are necessary for the transformation of BCR-ABL^+^cells, as well as surface molecules, which are necessary for cellular adhesion and interaction, giving advantages for cellular growth [161, 177]. In particular, the constitutive activation of NF*κ*B exists selectively in leukemia stem cells but not in normal HSC [178].

Alterations in NF*κ*B regulation and in the signaling pathways that control its activities are involved in cancer progression, as well as in the treatment resistance during chemo- and radiotherapy. NF*κ*B blocking can stop the proliferation of tumor cells or cause the tumor cells to become more sensitive to antitumor agents. This way, drugs that are capable of suppressing NF*κ*B activation have important therapeutic potential in the carcinogenesis inhibition [179]. Several studies have demonstrated that the expression of BCR-ABL kinase activity in CML cell lines leads to a constitutive activation of NF*κ*B through IKK*β* downstream of BCR-ABL and the suppression of NF*κ*B activation by the expression of I*κ*B*α* blocked BCR-ABL-dependent xenograft tumor formation [161–163].

Cilloni et al. [161] demonstrated that a selective inhibitor of the I*κ*B kinase (IKK) was capable of reducing NF*κ*B binding activity and proliferation, followed by induction of apoptosis in CML cell lines sensitive and resistant to imatinib, as well as in bone marrow cells from sensitive and resistant CML patients. Corroborating with these data, Duncan et al. [162] demonstrated that a selective IKK*β* inhibitor strongly suppressed growth and viability and induced cell death of cell lines expressing either wild-type or mutant versions of BCR-ABL, including the T315I mutation. Following the same rationale, Lounnas et al. [163] used another IKK*β* inhibitor to block NF*κ*B pathway capable of reducing cell survival and inducing apoptosis of imatinib-sensitive and imatinib-resistant cell lines. This work also demonstrated that cells from patients with T315I mutation appeared sensitive to NF*κ*B inhibition in terms of proliferation. Furthermore, *in vivo* experiments resulted in a significant regression of the tumors after the administration of the IKK*β*-inhibitor in nude mice injected with *BCR-ABL* wild-type and T315I mutant cells. Taken together, these results indicate that NF*κ*B/IKK is essential for BCR-ABL—induced cell growth and survival and that the kinase IKK*β* represents an attractive therapeutic target in CML.

Among these compounds acting as NF*κ*B inhibitors, proteasome inhibitors have been widely used. Recently, it has been shown that BCR-ABL induces the activity of the proteasome, supporting the idea of using the proteasome as a suitable target for BCR-ABL-expressing cells [180]. The proteasome inhibition results in the accumulation of I*κ*B in the cytoplasm, leading to inhibition of NF*κ*B translocation to the nucleus. The most used proteasome inhibitor is bortezomib/Velcade/PS341, inhibitor of the chymotrypsin-like activity of the *β*5 subunit of the proteasome. In several studies, proteasome inhibition induced proliferation arrest and apoptosis in imatinib-resistant cells, providing a rationale for the use of this drug in the subset of patients resistant to imatinib [164, 165]. Hu et al. [165] showed the combined effect of bortezomib and imatinib in CML. The combinatory regimens in CML murine models significantly reduced disseminated disease, decreased tumor growth, and induced apoptosis in tumor sections. In this work, the combination of bortezomib and imatinib repressed the DNA-binding activity of NF*κ*B. Albero et al. [164] demonstrated that bortezomib reduces proliferation and survival of Bcr-Abl- expressing cells, regardless of their sensitivity to imatinib, and including the highly resistant mutant T315I. In both studies, bortezomib inhibited proteasomal degradation of I*κ*B, leading to its accumulation. Taken together, these results suggest that an approach combining imatinib and proteasome inhibitors can be a therapeutic strategy in reducing relapse and overcoming imatinib resistance by inactivating the NF*κ*B pathway.

### 3.2. FoxO

FoxO transcription factors belong to the forkhead family of proteins, which are characterized by a conserved DNA-binding domain termed forkhead box (Fox) [181]. The FoxO class contains four members: FoxO1, FoxO3a, FoxO4, and FoxO6, whose expression can be found in a variety of different tissues [182]. FoxO proteins are implicated in crucial cellular functions including cell cycle regulation, stress response, glucose metabolism, and apoptosis [183]. Accumulating evidence suggests that FoxO act as tumor suppressors, inhibiting tumor growth by the activation of genes such as Bim, FasL, p27kip, cyclin D, GADD45a, glucose-9-phosphatase, and manganese dismutase [184]. Except for FoxO6, which is constitutively nuclear [185], phosphorylation by kinases, mainly Akt, ERK, (I*κ*B kinase) IKK, and serum and glucocorticoid-regulated kinase (SGK), regulates FoxO nuclear/cytoplasmic shuttling [186], leading to its nuclear exclusion, retention in the cytoplasm, and subsequent proteasome degradation and inactivation [187]. FoxO transcription factors can also be regulated by other posttranslational modifications such as acetylation, methylation, ubiquitination, and glycosylation [188].

Because BCR/ABL activity requires an activated PI3K/Akt pathway [189] and the inactivation of FoxO transcription factors was shown to be essential for tumorigenesis and resistance to treatment [190], the activation of FoxO by chemotherapeutic drugs seems to be a great strategy to overcome resistance [191]. Komatsu et al. [157] showed that BCR-ABL-positive cells have FoxO3a in a constitutively phosphorylated status and p27/kip1 downregulated. In agreement, exposure of CML cells to imatinib inhibited FoxO3a phosphorylation and induced p27/kip1 expression and G0/G1 arrest, blocking cell cycle progression. Essafi et al. [156] also showed that BCR-ABL inhibition induced by imatinib in CML cells resulted in FoxO3a activation. As a consequence, the induction of the FoxO3a-direct transcriptional target Bim was observed concomitantly with increased apoptosis. More recently, it was demonstrated that BCR-ABL-mediated FoxO3a inactivation was proteasome dependent [160]. Bortezomib treatment was able to restore FoxO3a expression, sensitize BCR-ABL T315I expressing cells to apoptosis, and inhibit CML-like disease in leukemic mice [160]. Regulation of FoxO3a expression affects the expression not only of Bim and p27/kip1, but also of cyclin D [155]. Imatinib-mediated inhibition of BCR-ABL represses cyclin D4 expression, upon FoxO3a activation and binding to cyclin D4 promoter. However, this effect can be prevented after FoxO3a silencing, indicating that FoxO3a is a key signaling molecule for BCR-ABL pathway and a relevant factor for apoptosis and cell cycle arrest in CML cells [155]. Imatinib can also exert its antileukemic effects through the concomitant activation of FoxO3a and the down-regulation of the inhibitor of DNA binding 1 (Id1) in K562 cells [159]. This study demonstrated that Id1 promoter is transcriptionally inhibited by FoxO3a, leading to differentiation of BCR-ABL transformed cells [159], suggesting that Id1 is essential for maintaining the leukemia phenotype. Moreover, experimental data suggest that FoxO3a activation can overcome imatinib resistance by increasing tumor necrosis factor-related apoptosis-inducing ligand (TRAIL) expression and by inducing apoptosis [158], further emphasizing the importance of the FoxO pathway in determining drug sensitivity.

 Although a great amount of evidence demonstrates that FoxO3a functions as a downstream factor for TKI-induced apoptosis, recent data suggest that FoxO3a has a crucial role in maintenance of CML stem cells. In a recent study, it was demonstrated that FoxO3a deficiency is associated with a decreased ability of leukemia-initiating cells (LICs) to provoke CML in FoxO3a^−/−^ mice [192]. Moreover, in CML stem cells, FoxO3a is predominantly nuclear and plays a resistant factor against TKI therapy [192]. Corroborating these data, the transcription factor Bcl-6 was identified as a target for the FoxO family, responsible for CML stem cells' self-renewal, repression of p53, leukemia initiation and resistance to TKI treatment [193]. As previously discussed [194], these findings reflect a “stem cell paradox” and may explain, in part, why CML stem cells persist after TKI treatment. The mechanisms and implications of these unexpected results regarding differential FoxO dynamics in CML stem cells still remain to be elucidated.

In conclusion, various findings have found that the activation of FoxO3a and its downstream genes are of clinical importance in diverse anticancer therapeutics, including in CML treatment. Different from p53 [17], FoxO mutation has not yet been found in human cancer, favoring FoxO targeted therapy. Clinical drugs which activate FoxO transcription factors can be used in combination with therapeutic agents for sensitizing CML malignant cells to therapy.

## 4. Molecular Interactions in Chemoresistance

**Figure 1:**
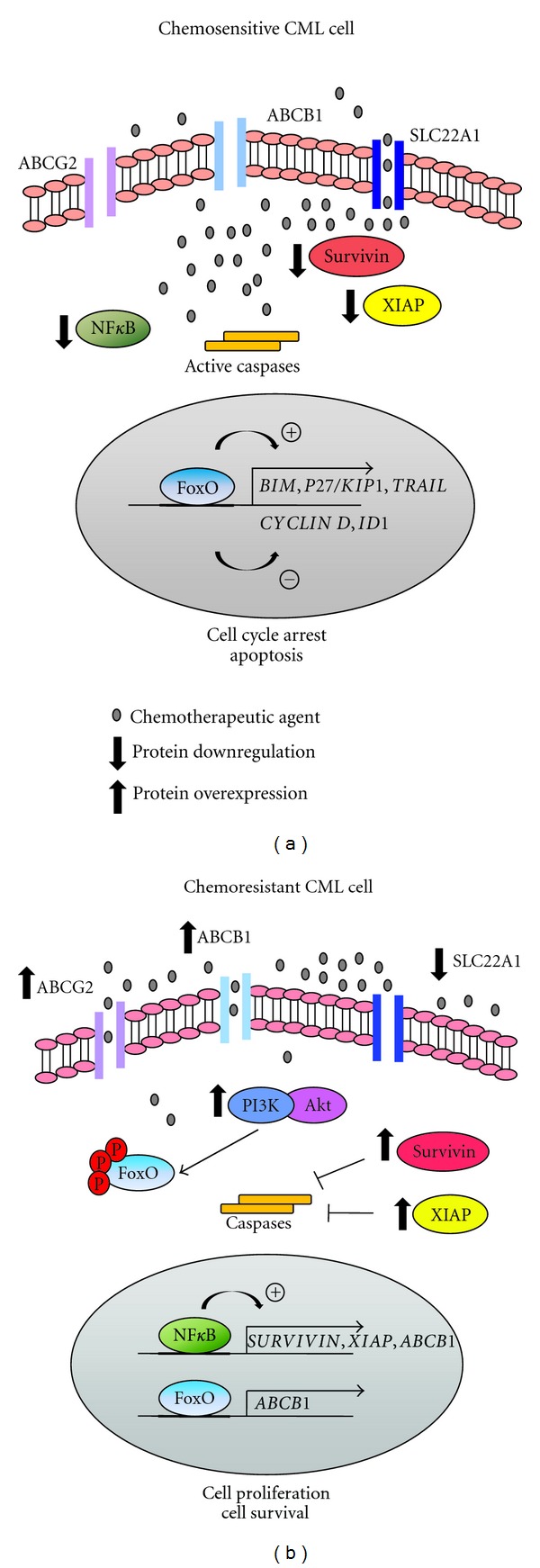
Molecular interactions in chemoresistance. Chemoresistant chronic myeloid leukemia (CML) cells display a multifactorial resistance phenotype characterized by deregulation of diverse signaling pathways which may act in concert or individually to prevent chemotherapy sensitivity (b). Resistant cells display constitutively active nuclear expression of NF*κ*B which contributes to stimulate transcription of the inhibitor of apoptosis proteins (IAPs) survivin and XIAP and also the efflux drug transporter ABCB1. The transcription factor FoxO3a, which usually acts as an apoptosis mediator, may also lead to enhanced ABCB1 transcription when chronically activated. In addition, chemoresistant CML cells display an overexpression of the efflux pump ABCG2 and reduced levels of the influx drug transporter SLC22A1. By contrast, many chemotherapeutic agents may overcome resistance and sensitize cells to apoptosis by modulating these pathways (a). Drug-mediated down-regulation of NF*κ*B, survivin, XIAP, and ABCB1 is associated with increased apoptotic levels, emphasizing their role as resistance factors. In addition, chemotherapy-induced FoxO3a activation results in cell cycle arrest and apoptosis by up-regulating *BIM, P27/KIP1, and TRAIL* and inhibiting *CYCLIN D* and *ID1* genes.

Growing evidence has demonstrated that the development of the MDR phenotype arises as a result of a complex network involving multiple cellular and molecular mechanisms. It is a multifactorial process rather than a consequence of a single and isolated mechanism (Figure 1). As the problem of drug resistance cannot be solved by circumventing only an individual protein, many efforts have been made in order to target diverse mechanisms and enhance cell sensitivity to antineoplastic therapy (Table 1).

Wang et al. [98] had suggested that clinical responses to imatinib treatment could be affected by transporters SLC22A1, ABCB1, and ABCG2; however, a recent work showed no significant differences between *ABCB1, ABCG2, and SLC22A1* genotypes and imatinib plasma or intracellular concentrations [195].

These data indicate that other transporters may be crucial for determining imatinib intracellular and plasma concentrations in CML patients. By contrast, in experiments using *in vitro* models of acquired resistance, K562 cells displayed upregulated levels of *ABCB1* and *ABCG2* genes, after exposure to increasing concentrations of imatinib [167, 168], which would imply the involvement of these transporters in resistance to TKIs [168]. However, different from the ABCG2 inhibitor, the ABCB1 inhibitor was able to restore imatinib sensitivity, indicating that only ABCB1 is essential for the development of acquired resistance in CML. Regarding the expression of SLC22A1 gene, contradictory data show that K562 resistant-cells had an increased [167] or similar [168] expression compared to their parental ones. Another work has demonstrated that imatinib and nilotinib are capable of inhibiting ABCB1 and ABCG2 and may overcome resistance, despite high levels of these transporters [63].

Current studies have proposed the role of IAPs in MDR phenotype promotion in association with ABCB1 expression [196]. Recently, we evaluated the resistance induced by the overexpression of both ABCB1 and survivin proteins [43]. In this work, we showed that K562 cells (ABCB1-negative) progressively became resistant to vincristine treatment by simultaneous overexpression of ABCB1 and survivin. We also showed that ABCB1 promoted resistance to cell death independently of its membrane expression. Besides that, we could observe that ABCB1 and survivin colocalize in the cytoplasmatic compartment, suggesting a common regulatory pathway of apoptosis resistance control [43]. In another work, we observed that both ABCB1 and survivin protein expressions are associated in CML patients [145]. We could establish a positive correlation between ABCB1 and survivin expression, but not with ABCB1 activity in samples from late-phase CML-CP patients. These data suggest that ABCB1 and survivin may act in promoting resistance in CML patients and, thus, reinforce the hypothesis that ABCB1 is able to induce resistance independently of its activity function [145]. As discussed above, CML patients usually develop imatinib resistance, and, therefore, new treatment approaches are necessary to overcome CML resistance. Netto et al. [197] showed that a new compound named LQB-118 was effective against leukemia cell lines with low toxicity to peripheral blood cells. Recently, we evaluated the effect of LQB-118 on CML cell lines and observed that this compound was able to induce apoptosis in both sensitive and resistant CML cells [166]. Moreover, cells treated with LQB-118 also presented decreased levels of survivin, XIAP, and ABCB1 expression. We also analyzed the LQB-118 effect in CML patient samples and observed that this compound was effective in inducing apoptosis in patients displaying the MDR phenotype [166]. Corroborating these data, Seca et al. [129] showed that the simultaneous inhibition of XIAP and ABCB1 in cells overexpressing ABCB1 could decrease imatinib resistance.

Recent studies reported that *ABCB1* expression can be regulated by the NF*κ*B transcription factor in hepatocytes and in drug-resistant cells. Moreover, the inhibition of NF*κ*B activity sensitizes resistant colon cancer cells through a decreased *ABCB1* expression, providing a link between NF*κ*B and resistance to chemotherapy through the regulation of human *ABCB1* gene expression [198]. In CML, Assef et al. [51] demonstrated that the resistance to imatinib exhibited in multidrug-resistant human leukemic K562 cells mediated by ABCB1 was reversed by the blockade of the NF*κ*B pathway using a specific NF*κ*B inhibitor [51]. Moreover, experimental evidence demonstrated the enhanced binding of NF*κ*B to the promoter region of *ABCB1* after K562 treatment with doxorubicin [199], further confirming the regulation of *ABCB1* by NF*κ*B in the promotion of chemoresistance. In accordance to that, FoxO3a may also interact with ABCB1 gene and decrease cell sensitivity. Some reports have postulated that chronic induction of Foxo3a expression and nuclear localization may activate mechanisms of resistance in CML cells. By using doxorubicin-sensitive and resistant K562 CML cells, Hui et al. [200, 201] have demonstrated that resistance to doxorubicin is associated with increased activity of PI3K/Akt, through a mechanism of feedback and with the *ABCB1* gene induction. In contrast, it was recently demonstrated that FoxO3a is able to inhibit survivin expression while inducing cell death in melanoma [202] and neuroblastoma-derived cell lines [203]. Moreover, FoxO3a and FoxO1 were able to physically interact and inhibit survivin promoter, confirming the interaction between FoxO transcription factors and the antiapoptotic protein survivin [204]. However, the interaction between survivin and FoxO proteins, and its role in imatinib sensitivity, has not been investigated yet in CML-derived cells.

Survivin can also be targeted by NF*κ*B [205], although it remains unclear how this interaction occurs. It was reported that inhibitors of the NF*κ*B pathway, such as the natural compounds triptolide [206] and berbamine [207], have been shown to induce apoptosis in CML imatinib-resistant cells by down-regulating survivin levels. XIAP is another identified NF*κ*B target, which is also implicated in modulating NF*κ*B activation, through a feedback loop mechanism, in response to DNA damage and bacterial infection [208]. Studies suggest that XIAP recruits TAK1 in order to achieve NF*κ*B activation and can mediate NF*κ*B activation by promoting degradation of COMMD1, a negative regulator of NF*κ*B [208]. As survivin, the interaction of XIAP and NF*κ*B in CML remains unclear.

## 5. Conclusions

Although the introduction of imatinib and other TKIs in CML therapy has brought improvements in survival, CML prognosis still remains unfavorable for a group of patients. In addition to mutations found in the *BCR-ABL* gene, which alter the BCR-ABL kinase domain, there are currently identified secondary mechanisms of TKIs resistance. Multiple factors, such as inhibition of apoptotic signaling pathways, reduction in drug accumulation, and alterations in transcription factors, are known to contribute to the development of MDR and treatment failure in CML. These mechanisms usually act in concert in a multifactorial resistance context and play their role independent of or downstream BCR-ABL tyrosine kinase. Because the inhibition of only one mechanism is not effective enough to overcome clinical TKIs resistance, suppressing simultaneously several proteins must be required to increase the efficacy of the treatment in CML patients. Several questions remain to be answered to understand the interplay between these modes of resistance. For instance, how these proteins interact with each other to promote resistance and which one must be completely suppressed to antagonize malignancy? Regardless, what we know is that chemoresistance in CML is a multifactorial phenomenon and targeting these molecules seems to represent an interesting and feasible approach to overcome the development of TKIs-resistance in CML.

## Figures and Tables

**Table 1:** Anticancer drugs sensitize CML cells by targeting IAPs, drug transporters, NF*κ*B and FoxO proteins.

Drug or therapy	Protein(s) targeted	Signaling pathways affected
Imatinib, idarubicin	Survivin	Imatinib and idarubicin inhibited viability and induced apoptosis in cells derived from a Ph^+^ patient in blast crisis and K562 cells, respectively, through survivin downregulation [144].
Imatinib	Survivin	Enhanced imatinib-mediated apoptosis by modulating reactive oxygen species [147] and using antisense oligonucleotide or dominant-negative survivin [154] in CML cell lines.
Microtubule stabilizing agents and flavopiridol vorinostat, MK0457	Survivin	The combination of microtubule stabilizing agents and the cyclin-dependent kinase inhibitor flavopiridol [149] as well as the cotreatment with vorinostat and the aurora kinase inhibitor [155] led to survivin inhibition and increased apoptosis levels in K562 cells.
Sheperdin	Survivin	The survivin inhibitor molecule showed great toxicity against CML and AML cells, with no decrease in viability of phytohemagglutinin-stimulated peripheral blood mononuclear cells [153].
Imatinib	FoxO3a	Imatinib-mediated BCR-ABL inhibition resulted in FoxO3a activation, induction of Bim [156], p27/kip1 [157] and tumor-necrosis-factor-related apoptosis-inducing ligand (TRAIL) [158], repression of cyclin D4 expression [156] and inhibitor of DNA binding 1 (Id1) [159], and consequent increased apoptosis in CML cell lines.
Bortezomib	FoxO3a	Bortezomib treatment was able to restore FoxO3a expression, sensitize imatinib-resistant T315I expressing cells to apoptosis, and inhibit CML-like disease in leukemic mice [160].
IKKB inhibitors	NF*κ*B	The IKKB inhibitors led to the induction of apoptosis in cell lines (K562 and KCL) and bone marrow cells sensitive and resistant to imatinib [161], induced cell death in cell lines BaF3 BCR-ABL wild-type or mutant, including T315I mutation [162], suppressed proliferation of cells from patients with T315I mutation and *in vivo *experiments resulted in a regression of the tumors in nude mice [163].
Bortezomib	NF*κ*B	Bortezomib reduced proliferation and survival of BCR-ABL-expressing cells, regardless of their sensitivity to imatinib and including the mutant T315I [164], and the combinatory effect with imatinib in CML led to reduced disseminated disease, decreased tumor growth and induced apoptosis in tumor sections [165].
Vincristine	ABCB1 and survivin	Overexpression of ABCB1 and survivin were associated with low apoptosis index induced by vincristine treatment [43].
LQB-118	ABCB1, survivin and XIAP	LQB-118 overcome resistance phenotype through ABCB1, survivin and XIAP downregulation [166].
Imatinib and nilotinib	*SLC22A1*, *ABCB1* and *ABCG2 *	K562 cells displayed upregulated levels of *SLC22A*1, *ABCB1,* and *ABCG2* genes, after exposure to increasing concentrations of imatinib and nilotinib, respectively [167].
Imatinib	SLC22A1, ABCB1 and ABCG2	Chronic exposure to imatinib increased ABCB1 and ABCG2 at the protein and gene levels, but SLC22A1 expression remained unaltered [168].
Imatinib and vincristine	XIAP and ABCB1	Simultaneous inhibition of XIAP and ABCB1 in cells that overexpress this efflux pump decreases the resistance to imatinib [129] and vincristine [130].
Imatinib, apicidin and EBT-737	XIAP	Imatinib-induced apoptosis was found to be associated with XIAP downregulation [121] and could be potentiated when combined with apicidin [122] and EBT-737 [123] in K562 cells and CML progenitors.
Etoposide and doxorubicin	XIAP	The downregulation of XIAP expression with antisense oligonucleotides increased apoptosis and enhanced the effects of doxorubicin in K562 cells [128].

AML: acute myeloid leukemia, CML: chronic myeloid leukemia; IAPs: inhibitor apoptosis proteins.

## Abbreviations

ALL: Acute lymphoid leukemia
AML: Acute myeloid leukemia
BCR-ABL: Breakpoint cluster region/V-abl Abelson murine leukemia viral oncogene homolog 1
BCRP: Breast-cancer-related protein
BIR: Baculoviral IAP repeat
BP: Blast phase of chronic myeloid leukemia
CDK1: Cyclin-dependent kinase 1
CLL: Chronic lymphoid leukemia
CML: Chronic myeloid leukemia
FOX: Forkhead box
HSC: Hematopoietic stem cell
IAP: Inhibitor of apoptosis proteins
Id1: Inhibitor of DNA binding 1
I*κ*B: Inhibitor of NF*κ*B
IKK: I*κ*B kinase
KD: Kinase domain
LIC: Leukemia initiating cells
MAPK: Mitogen-activated protein kinase
MDR: Multidrug resistance
MRP1: Multidrug resistance protein 1
MTA: Microtubule targeting agents
NF*κ*B: Nuclear factor kappa B
OCT-1: Organic cation transporter-1
Pgp: P-glycoprotein
Ph: Philadelphia
SGK: Serum and glucocorticoid-regulated kinase
Si-RNA: Small interfering RNA
SP: Side population
TKI: Tyrosine kinase inhibitors
TRAIL: Tumor-necrosis-factor-related apoptosis-inducing ligand
XIAP: X-linked of inhibitor of apoptosis protein.
